# The Cytoplasm-to-Vacuole Targeting Pathway: A Historical Perspective

**DOI:** 10.1155/2012/142634

**Published:** 2012-02-20

**Authors:** Midori Umekawa, Daniel J. Klionsky

**Affiliations:** Life Sciences Institute, University of Michigan, Ann Arbor, MI 48109-2216, USA

## Abstract

From today's perspective, it is obvious that macroautophagy (hereafter autophagy) is an important pathway that is connected to a range of developmental and physiological processes. This viewpoint, however, is relatively recent, coinciding with the molecular identification of autophagy-related (Atg) components that function as the protein machinery that drives the dynamic membrane events of autophagy. It may be difficult, especially for scientists new to this area of research, to appreciate that the field of autophagy long existed as a “backwater” topic that attracted little interest or attention. Paralleling the development of the autophagy field was the identification and analysis of the cytoplasm-to-vacuole targeting (Cvt) pathway, the only characterized biosynthetic route that utilizes the Atg proteins. Here, we relate some of the initial history, including some never-before-revealed facts, of the analysis of the Cvt pathway and the convergence of those studies with autophagy.

## 1. The Background

To understand the origin of the studies that led to the identification of the Cvt pathway, we need to briefly step back into the early days of yeast molecular genetics. Randy Schekman's group was studying the secretory pathway and isolating mutants defective in various steps including endoplasmic reticulum (ER)-to-Golgi transport as well as secretion to the cell surface. Two former postdocs from the Schekman lab, Scott Emr and Tom Stevens, decided to pursue a similar direction, but to avoid a direct overlap with Randy Schekman by focusing on a pathway that branches off from the secretory pathway, the delivery of proteins to the vacuole. The Emr and Stevens labs isolated a new set of mutants initially named *vpt* (vacuolar protein targeting) [1] and *vpl *(vacuolar protein localization) [2], and subsequently *vps* (vacuolar protein sorting), which are defective in the delivery of resident proteins to the vacuole. Being interested in protein sorting, one of us (D.J.K.) went to Scott Emr's lab to learn about yeast.

While in the Emr lab, I characterized the vacuolar delivery of proteinase A (Pep4) and vacuolar alkaline phosphatase (Pho8). Around that time, the sequence of the gene encoding another vacuolar hydrolase, aminopeptidase I (Ape1) was published [3, 4]. It is important to keep in mind that this was the late 1980s, quite some time before the *Saccharomyces cerevisiae* genome was sequenced in its entirety. In fact, automated sequencing was relatively new, so it was still a major accomplishment when a gene was sequenced. Until then, only the sequences of Pep4 [5, 6], Prc1 (carboxypeptidase Y) [7], Pho8 [8], Prb1 (proteinase B) [9], and Ams1 (*α*-mannosidase) [10] were known among the vacuolar hydrolases. Thus, it was quite exciting to those of us studying vacuolar protein targeting when a new protein sequence became available. One of my main goals in the Emr lab was to identify the vacuolar-targeting motif and determine a consensus sequence (mapping consensus targeting or retention signals was very popular in those days), a task that was all the more difficult due to the limited number of proteins available for comparison. Hence, I was particularly interested in having a new protein that I could analyze.

Ape1 was known to be a vacuolar hydrolase, and it was characterized as being a glycoprotein [11]. The latter finding fit with the fact that all of the characterized vacuolar hydrolases traffic through the secretory pathway to the Golgi complex and from there are diverted to the vacuole. One interesting feature of the protein sequence for the precursor form of Ape1 (prApe1), however, was that it lacked a standard signal sequence. Accordingly, I assumed that it entered the ER by a unique mechanism. This seemed to add some additional interest to the analysis, as the idea of analyzing yet one more vacuolar hydrolase was getting somewhat tedious. When I discussed the idea of analyzing the targeting of prApe1 with Scott Emr, however, he was not interested. After all, even if the details of the process were slightly unusual, we were still talking about the characterization of another vacuolar hydrolase that transits through a portion of the secretory pathway. Indeed, at the time, there seemed to be more interesting projects to pursue, so the analysis of prApe1 was left on the “back burner”.

Shortly after that time, I started an independent position at the University of California, Davis. To stay clear of the Emr lab (which, for a new assistant professor, loomed like an 800-pound gorilla), I pursued an analysis of the vacuolar H^+^-translocating ATPase and vacuolar acid trehalase. At that time, Scott forwarded to me a letter (this was just before email became widely used) from a postdoc applicant that he was not able to invite to his lab. That postdoc, Nieves Garcia Alvarez, was from one of the labs, that of Paz Suarez-Rendueles, which was involved in characterizing yeast vacuolar hydrolases, and I agreed to offer her a position. Nieves initially worked on the vacuolar ATPase project. I knew, however, that her lab in Spain was one of two that had essentially simultaneously sequenced the *APE1/LAP4* gene encoding prApe1 [4]. During Nieves' time in my lab, I wrote to Beth Jones who had published one paper on Ape1 [12] and asked if she intended to pursue this topic; I did not want to compete with her, but she indicated that she was not going to be working on it, and I was welcome to it. Thus, I obtained the gene from the Suarez-Rendueles lab and a new postdoc from that lab, Rosaria Cueva Noval, along with my postdoc Debbie Yaver and me, began to examine the vacuolar targeting of prApe1.

The initial experiments on prApe1 were confusing, because I could not find any evidence for glycosylation or for the existence of the protein within the compartments of the secretory pathway [13]. (As a side note, our first paper on Ape1 was published back-to-back with the first paper from Yoshinori Ohsumi's lab on the characterization of autophagy in yeast [14]. This was coincidental, and, to be honest, I paid no attention to the Ohsumi paper at that time, because it was on the topic of autophagy; I was studying protein targeting, not some presumed “garbage” pathway that was only used for protein degradation.) Eventually, it dawned on me that the published data were incorrect and that Ape1 was not a glycoprotein. At this time, Fred Dice was making headlines with his analysis of the KFERQ—(KFERQ being the consensus sequence for the recognized substrates) or pentapeptide-dependent pathway for the transport of proteins into the lysosome (the current name for this pathway, “chaperone-mediated autophagy,” had not been coined yet) [15]. Considering that Ape1 was not a glycoprotein, and that it did not enter the endoplasmic reticulum, I reasoned that it entered the vacuole by translocating directly across the limiting membrane. Accordingly, I further assumed that there must be protein machinery, similar to the as yet uncharacterized components involved in the KFERQ pathway, in the vacuolar membrane just waiting for me to come along and identify them.

Therefore, in order to identify the vacuolar membrane translocation components, we generated a chimera of prApe1 fused to the *HIS3* gene. Our initial screen was based on the idea that a *his3* mutant strain of yeast would not be able to grow in the absence of histidine if the chimera was efficiently delivered to the vacuole. Accordingly, we could isolate mutants that were able to grow without histidine, and they would have defects in the various components of the translocation machinery. It became clear early on that the screen was not working, although we did not know why; we could not easily follow the localization of the chimera because the green fluorescent protein was not yet being used for cell biology studies. Randy Schekman was giving a seminar on campus at that time, and I told him about our project. He suggested that we generate antibodies that only recognized prApe1 and carry out a screen looking for mutants that accumulate the precursor form of the protein. We did attempt that approach, using colony blots after transferring cells to nitrocellulose, but it was very difficult to score positive colonies. However, we also noticed that wild-type cells analyzed by western blot, when grown appropriately, had essentially no prApe1; all of the protein was in the mature form. We also determined (using a *pep*4Δ mutant as the control) that we could easily detect the precursor that accumulated when one out of ten colonies was defective for prApe1 maturation. Accordingly, even though it was laborious, Tanya Harding, and later Ann Hefner-Gravink, in my lab began to analyze random mutants in batches of ten for the accumulation of prApe1.

We isolated a series of such mutants and placed them into complementation groups [16]. This was quite exciting as we were finally about to identify the long-awaited translocation machinery for the vacuole. To be sure that we were not going to waste our time analyzing mutants that were already known, we began to compare our mutants with all other previously identified mutants that affected vacuolar protein delivery. Of course this included the *vps* mutants from Tom Stevens and Scott Emr, but also endocytosis mutants and vacuolar morphology (*vam*) mutants. Even though we did not expect overlaps from the latter, we wanted to be thorough. In fact, we were so careful that we even requested protein extracts from Yoshinori Ohsumi and Michael Thumm, who had isolated *apg* [17] and *aut* [18] mutants, respectively, that are defective in autophagy. Obviously (or so we thought at the time), there was not going to be an overlap; autophagy is a degradative pathway, and our mutants (then named *cvt*) were defective in a biosynthetic pathway. Imagine our surprise, and disappointment, when we found an essentially complete overlap among these three sets of genes [19, 20]. The disappointment was for two reasons. First, instead of having a unique set of mutants that we could study on our own, we knew we immediately had competitors. Second, we were being dragged against our will into the field of autophagy.

**Figure 1:**
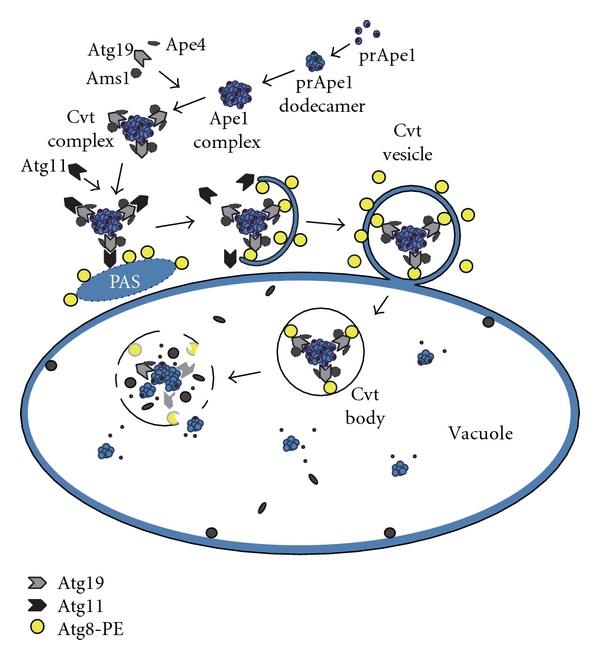
Overview of the Cvt pathway. (1) Formation of the Cvt complex: Precursor Ape1 forms a dodecamer. Multiple dodecamers assemble into an Ape1 complex. The Ape1 complex binds Atg19 via the prApe1 propeptide to form the Cvt complex. Other Cvt cargo, including Ams1 and Ape4, bind Atg19 at distinct domains. (2) Movement to the PAS: Atg19 binds the scaffold protein Atg11, and the Cvt complex moves to the PAS. (3) Formation of the Cvt vesicle: Atg19 binds Atg8–PE, which drives the sequestration of the Cvt complex by the double-membrane phagophore. (4) Fusion of the Cvt vesicle with the vacuole: After completion of the Cvt vesicle, the outer membrane fuses with the vacuole, releasing the single membrane Cvt body into the lumen. The Cvt body is broken down by the Atg15 lipase, allowing access to vacuolar hydrolases. Atg19 and Atg8 are degraded. The propeptide of prApe1 is removed and the enzyme becomes active.

Nonetheless, we continued with our studies of prApe1 targeting and began to clone the *CVT/APG/AUT* genes and analyze the gene products. After discovering the overlap with the *APG* genes, we sent purified antisera against Ape1 to the Ohsumi lab to be used in an electron microscopy analysis by Misuzu Baba. I can still remember Yoshinori Ohsumi cryptically telling me about some striking and exciting results that “could not be described” over the phone, but that had to be seen in person. This resulted in a visit to Japan, and the viewing of images that were indeed striking, revealing that prApe1 import was morphologically similar to autophagy (Figure 1) [21]. Much of the initial work on the characterization of the Atg proteins was done in collaboration with the Ohsumi lab [20, 22–27] and also with the lab of Bill Dunn [22, 28–32], who was studying peroxisome degradation in *Pichia pastoris*. Having established the historical perspective, we now present some of the details of those initial studies of the Cvt pathway, starting with the characterization of aminopeptidase I import by a mechanism that is independent of the secretory pathway, identification of the vacuolar targeting domain, the isolation of mutants defective in prApe1 delivery to the vacuole, and concluding with the genetic and morphological studies that revealed the overlap with autophagy.

## 2. The Transport of prApe1 to the Vacuole Is Mediated by the Cvt Pathway

Ape1 was initially characterized as a vacuolar enzyme that hydrolyzes leucine peptides (hence the original nomenclature leucine aminopeptidase, or LAP, which is unfortunately confusing because LAPI is encoded by the *LAP4* gene, whereas LAPIV is encoded by *LAP2*, etc.) [33]. The hydrolase is synthesized as an inactive zymogen containing a propeptide that may sterically block its active site; it is processed to its mature form in the vacuole by proteinase B in a *PEP4*-dependent manner [34]. As mentioned above, published data suggested that the Ape1 precursor was transported through part of the secretory pathway, because it was characterized as a glycoprotein [11]. However, a detailed characterization of prApe1 biosynthesis suggested that its delivery to the vacuole was independent of the secretory pathway: (1) prApe1 lacks a signal sequence for transport into the ER, and it is not glycosylated; (2) the half-life of processing (i.e., removal of the propeptide in the vacuole) of prApe1 is substantially longer (~30 min) than that of Prc1 or Pep4 (~6 min), both of which are transported to the vacuole via part of the secretory pathway; (3) vacuolar import of prApe1 is relatively unaffected by *sec* mutants [13].

The obvious question then became, how does prApe1 target to and enter the vacuole? A series of biochemical analyses were performed to address this issue. After it is synthesized as a 61-kDa protein in the cytosol, prApe1 is proteolytically processed to a mature 50-kDa form in the vacuole. The prApe1 propeptide plays an essential role in the transport process [35]. A detailed mutagenesis analysis carried out by Mike Oda revealed that the first amphipathic *α*-helix in the propeptide is critical for the vacuolar targeting of the enzyme. Deletion of the precursor region or mutations that affect the first *α*-helical region inhibit its binding to the membrane fraction and prevent subsequent vacuolar delivery and processing. Further analysis by John Kim revealed that prApe1 is assembled as a dodecamer (~669 kDa) in the cytoplasm prior to vacuolar delivery, which argued against direct translocation across the vacuole limiting membrane [36]. The propeptide of prApe1 is not required for its oligomerization. A pulse chase analysis showed that the oligomeric assembly and the subsequent membrane association are very rapid events with a half-life of ~3 min. These results suggested that the long half-life of prApe1 transport may be due to the rate limiting step of the import of the dodecameric enzyme into the vacuole lumen after its binding to membrane.

## 3. The Cvt and Autophagy Pathways Share the Same Machinery

The oligomerization of prApe1 and the slow kinetics of import into the vacuole argued against transport through the secretory pathway. To understand the mechanism of vacuolar delivery, a detailed biochemical and genetic analysis was carried out in *S. cerevisiae*, which revealed that autophagy and the Cvt pathway largely share the same machinery for double-membrane vesicle formation [16, 19, 20, 27]. A genetic screen to analyze the Cvt pathway was carried out by monitoring the accumulation of prApe1 as described in Section 1. From the initial screen, five *cvt* mutants (*cvt2/atg7, cvt3, cvt5/atg8, cvt6 and cvt7/atg9*) were isolated, which showed a complete block in prApe1 processing, but were not defective in the maturation of the precursor form of Prc1 or Pep4 [16]. Most of these mutants also showed a defect in nonselective autophagy [19, 20]. Just prior to the isolation of the *cvt* mutants, Michael Thumm in Dieter Wolf's lab isolated a series of *aut* mutants, based on defects in the degradation of the fatty acid synthase. The *aut* mutants including *aut3* (*cvt10/atg1*), *aut5* (*cvt17/atg15*), *aut7* (*cvt5/atg8)*, and *aut9* (*cvt7/atg9*) also displayed a significant block in the maturation of prApe1, providing genetic evidence for a role of these proteins in both the Cvt pathway and autophagy [19]. A similar analysis of the *apg* mutants from Yoshinori Ohsumi's lab also revealed an extensive overlap [20]. Subsequently, all of the *ATG* genes, except *ATG11*, *ATG17*, *ATG19*, *ATG22*, *ATG29*, and *ATG31* were found to be required for both pathways. In 2003, the nomenclature for these *CVT* and *APG/AUT* genes was unified as “*ATG*” for “autophagy related” [37].

## 4. Precursor Aminopeptidase I Is Imported by a Vesicular Mechanism

The genetic overlap between the *cvt* and *apg/aut* mutants gave rise to the idea of a vesicle-mediated mechanism for prApe1 import. Indeed, electron microscopy analyses performed by Misuzu Baba revealed that the prApe1 dodecamers further assembled into a large complex composed of multiple dodecamers (called an Ape1 complex), and that in the cytoplasm this complex is surrounded by a double membrane-bound structure, followed by fusion with the vacuolar membrane [21], similar to what was observed in bulk autophagy [38]. This result demonstrated the use of an autophagy-like mechanism for the Cvt pathway. However, the double membrane structure enwrapping the Ape1 complex (termed a Cvt vesicle) is ~150-nm in diameter, in contrast with that of the autophagosome, which is 300–900 nm. In addition, the Cvt vesicle, in contrast to the autophagosome, excludes bulk cytoplasm. Furthermore, while autophagy is induced under starvation conditions, the Cvt pathway occurs constitutively in growing conditions. Finally, as we mentioned above, the Cvt pathway is a selective, biosynthetic pathway, whereas autophagy is generally nonselective and is degradative. How then could we explain the apparent overlap in the import machinery? Importantly, when cells are subjected to starvation, the Cvt complex is sequestered within a larger autophagosome [38], although the kinetics for import are essentially the same as during vegetative growth. Thus, while the biosynthetic Cvt pathway can be distinguished from autophagy, the Ape1 complex can be taken up by autophagosomes under starvation conditions, again suggesting that the Cvt pathway and autophagy utilize much of the same machinery.

In *S. cerevisiae*, the biogenesis and the vacuolar transport of both autophagosomes and Cvt vesicles include the following steps: (1) membrane from various sources generates vesicles containing Atg9 (see below) as a critical integral membrane protein, and these vesicles form into tubulovesicular clusters in a SNARE-dependent manner; (2) one or more clusters contribute to the formation of a perivacuolar phagophore assembly site (PAS), which is considered to be a foundation/nucleation site that (3) leads to formation of the phagophore, the initial sequestering compartment; (4) two ubiquitin-like protein conjugation systems including Atg8 and its conjugation to phosphatidylethanolamine (PE) contribute to the formation and elongation of the phagophore to generate the double-membrane Cvt vesicle and autophagosome; (5) the completed vesicles dock and fuse with the vacuole, releasing the inner vesicle into the lumen where the single-membrane structures are referred to as Cvt or autophagic bodies.

Both autophagosomes and Cvt vesicles are said to be formed *de novo*, to emphasize the fact that their generation occurs by a mechanism that is distinct from that used in the budding of transient transport vesicles in the secretory pathway. Although the details of sequestering vesicle biogenesis are still not clear, almost all of the Atg proteins are localized at least transiently to the PAS [39]. Atg9, which is the sole integral membrane protein in yeast that is essential for Cvt vesicle and autophagosome formation, is relatively unique in that it is localized at multiple sites including the PAS. The population of Atg9 at the non-PAS sites (Atg9 reservoirs) corresponds to the tubulovesicular clusters and is proposed to traffic between these sites and the PAS, providing membrane for phagophore expansion. The function of most of the Atg proteins is still not known. For example, Atg8–PE participates in cargo recognition during selective types of autophagy and is also involved in determining the size of the autophagosome [40], but the details of these mechanisms are not known.

## 5. Discovery of the Cvt-Specific Genes

As mentioned above, not all of the *cvt* and *apg/aut* mutants displayed an overlap; some mutants were defective only in autophagy or the Cvt pathway, but not both. For example, the *atg11* mutant shows a complete block in the maturation of prApe1, but is essentially normal for autophagy [19, 23]. These results suggested that the Cvt pathway and autophagy share most of the same machinery, but that they also need some molecules that are specific for each pathway. One of the fundamental differences between the Cvt pathway and autophagy concerns their temporal and physiological activity. The Cvt pathway is active during vegetative growth, consistent with its role as a biosynthetic trafficking route. In contrast, autophagy is induced under starvation conditions, where it can break down cellular macromolecules to supply building blocks and energy. A complex of proteins including Atg13, which is required both for the Cvt pathway and autophagy, appears to be partly responsible for switching these pathways in response to changes in the environment. In starvation conditions, Atg13 interacts with the Atg1 complex including Atg17, Atg29, and Atg31 to induce autophagy [22, 41–43]. Under vegetative conditions, Atg13 may have a lower affinity for Atg1, a condition that may promote the Cvt pathway. Atg13 is regulated by its phosphorylation status in a TORC1-dependent manner; Atg13 is highly phosphorylated in growing conditions but dephosphorylated in starvation conditions [41, 44].

Another characteristic of the Cvt pathway is the specificity for its cargo, whereas macroautophagy is a nonselective process, suggesting that the Cvt pathway requires a receptor, which recognizes the substrate. In this case, the substrate corresponds to the cargo of the Cvt vesicles, which is comprised primarily of the Ape1 complex. A systematic yeast two-hybrid screen in *S. cerevisiae* was performed and the gene product of *YOL082W* was found as a potential interacting protein with prApe1 [45]. Biochemical analysis demonstrated that *YOL082W* encodes a protein that functions as a receptor for the targeting of prApe1 by the Cvt pathway, and the gene was renamed *CVT19 *[46, 47] and later *ATG19* [37]. In *atg19Δ* cells, the precursor form of Ape1 accumulates in the cytoplasm in both nutrient rich and starvation conditions, suggesting that Atg19 is necessary for the targeting of prApe1 both by the Cvt pathway and autophagy. An important point in this regard is that import of prApe1 by autophagy is still a selective process that utilizes a receptor protein; this explains why the kinetics of import are the same as for the Cvt pathway and are much faster than would be expected for bulk uptake of cytoplasm.

An immunoprecipitation analysis showed that Atg19 physically interacts with the propeptide of prApe1, and the coiled-coil domain of Atg19 mediates this interaction [48]. Atg19 localizes at the PAS with the Ape1 complex [49]; the combination of the Ape1 complex bound to Atg19 is referred to as the Cvt complex. In *atg19Δ *cells, GFP-Ape1 forms a dodecamer, but it does not localize at the PAS. The kinetics of the maturation of prApe1 and the degradation of Atg19 are quite similar. Together with the localization data, these findings suggest that Atg19 is delivered to the vacuole by the Cvt pathway along with the precursor Ape1 dodecamer. Interestingly, deletion of *APE1* results in a dispersed Atg19 distribution, and Atg19 does not localize to the PAS in *ape1Δ* cells, suggesting that the Ape1 complex itself is required for concentrating its soluble receptor at this site. Further analyses revealed that Atg19-prApe1 movement to the PAS is dependent on Atg11, which we now know acts as an adaptor or scaffold protein for selective autophagy pathways, such as the Cvt pathway, and the selective autophagic degradation of peroxisomes and mitochondria (termed pexophagy and mitophagy, resp.) [22, 50]. Atg11 may mediate the transport of Atg9 to the PAS for selective autophagy during vegetative growth [51], whereas Atg17 may carry out this role for bulk autophagy during starvation. Atg11 has certain characteristics of a scaffold protein in that it interacts with several Atg proteins, including Atg1, Atg9, Atg17, Atg19, Atg20, and itself [51, 52].

In the Cvt pathway, Atg19 binds the prApe1 propeptide independent of any other Atg proteins. Atg11 can then interact with Atg19, allowing movement of the cargo to the PAS. Once at the PAS, Atg19 also interacts with Atg8–PE; it is not known if both Atg8 and Atg11 bind Atg19 at the same time, as their binding sites are distinct, but very close to each other. Thus, Atg19 is a receptor that is responsible for recognizing the prApe1 dodecamer to target it to the PAS due to its interaction with Atg11. Furthermore, Atg19 leads to the incorporation of the Cvt complex into a double-membrane vesicle (i.e., a Cvt vesicle or autophagosome) via its interaction with Atg8 [48]. In the absence of other Atg proteins such as Atg1, Cvt vesicles, and autophagosomes do not form; however, the Cvt complex is still targeted to the PAS, suggesting that Atg19 transport of prApe1 to the PAS occurs independent of the vesicle formation steps. Atg19 is both ubiquitinated and deubiquitinated *in vivo*, and these modifications of Atg19 are required for the efficient trafficking of prApe1 via the Cvt pathway [53]. Atg19 interacts with the deubiquitinating enzyme Ubp3, and the deletion of *UBP3* leads to decreased targeting of prApe1. Furthermore, the mutation on the ubiquitin acceptor site, Lys213 and Lys216 of Atg19, reduces the interaction of Atg19 with prApe1. Thus, the ubiquitination and deubiquitination of Atg19 are likely to play a structural or mechanistic role in the normal progression of the Cvt pathway, instead of serving as a degradation signal for the proteasome.

As described above, many of the yeast Atg proteins responsible for the Cvt pathway and autophagy have been identified, and the general mechanism involved in these processes has been explored through genetic and biochemical approaches. Nevertheless, the molecular mechanism underlying nucleation of the sequestering phagophore remains largely unknown. Many processes involving membrane rearrangement and movement, such as endocytosis or membrane ruffling, require the cytoskeleton. The actin cytoskeleton is required for the selective Cvt pathway, but not for nonselective autophagy in yeast [54]. Actin plays a role in trafficking of Atg9 to the PAS and recruitment of the Cvt cargo in growing conditions. Further studies identified actin-related proteins, including components of the Arp2/3 complex, as playing a role in the transport of Atg9 for specific types of autophagy [55]. The Arp2 protein itself interacts with Atg9 and regulates the dynamics of Atg9 movement. Thus, the Arp2/3 complex may allow Atg9, along with its associated membrane, to move in a directed fashion to the PAS along actin cables. The specific autophagy factors such as Atg19 and Atg11, and perhaps other molecular components, may serve as adaptors between the Cvt cargo and the actin cytoskeleton.

## 6. Discovery of Other Cvt Cargo, Ams1 and Ape4

Prior to the analysis of the Cvt pathway, Ams1 was shown to enter the vacuole independent of the secretory pathway [56], although the mechanism of import was unclear. We found that Ams1 is another hydrolase targeted to the vacuole by the Cvt pathway [57], as its delivery is blocked in *cvt* (*atg*) mutants. Similar to prApe1, Ams1 forms oligomers composed of 4 to 6 of the 122-kDa species in the cytosol, and the oligomeric state is maintained during the import process. Ams1 transport is also mediated by Atg19 [47] and its binding site is distinct from that used by prApe1 [48]. Thus, Ams1 is part of a prApe1-Atg19-Ams1 Cvt complex. In *ape1Δ *cells, the Ams1-Atg19 interaction still occurs, but this complex is dispersed in the cytosol, whereas deletion of *AMS1* does not affect the transport of the prApe1-Atg19 complex. These results indicate that Ams1, which is synthesized at a level that is substantially lower than prApe1, might exploit the prApe1-Atg19 import system to achieve its own efficient transport to the vacuole.

Recently, it was shown that Ams1 is delivered to the vacuole in an Atg19-independent manner under starvation conditions [58]. During autophagy, Atg34 (Yol083w), a homolog of Atg19, functions as a receptor for Ams1. In *atg19Δ* cells, Ams1 targeting is disrupted in nutrient-rich conditions [47, 48]. However, Ams1 is efficiently transported into the vacuole under starvation conditions by autophagy even in *atg19Δ *cells. A genome-wide yeast two-hybrid screen suggested that Yol083w is an Ams1 interacting protein [45], and Atg34 indeed physically interacts with Ams1 [58]. Similar to Atg19, Atg34 binds Atg8 and Atg11 using distinct domains, and these interactions are essential for its function in targeting Ams1 into an autophagosome; an Atg34 mutant that lacks its Atg8 interacting motif forms a complex with Ams1, but shows a defect in sequestration into autophagosomes. Importantly, the transport of Ams1 mediated by Atg34 in starvation conditions is prApe1 independent, unlike that mediated by Atg19 in growing conditions.

Also recently, aspartyl aminopeptidase (Yhr113w/Ape4) was found to be a third Cvt cargo protein [59]. Yeast two-hybrid analyses suggested that Ape4 can associate with Atg19 and prApe1 [60]. Unlike prApe1, Ape4 does not possess a propeptide region and it does not self-assemble into aggregates [59]; however, it still binds to Atg19. An immunoprecipitation analysis with truncated versions of Atg19 revealed that the three identified Cvt cargo components, prApe1, Ams1, and Ape4, associate with Atg19 by binding to distinct sites. GFP-Ape4 colocalizes with RFP-Ape1 at the PAS in growing conditions, and this localization is dependent on Atg19. Notably, Ape4 transport to the vacuole by the Cvt pathway is significantly decreased in *ape1Δ* cells, suggesting that Ape4 relies on the prApe1-Atg19 complex for its targeting, similar to Ams1 in vegetative conditions. In *atg11Δ *cells, Ape4 can colocalize with prApe1, but it does not localize at the PAS.

## 7. Conclusions

An intriguing question has been why yeast cells have utilized the Cvt pathway to import a resident vacuolar hydrolase. In higher eukaryotes, there is no evidence for a Cvt pathway, and the *ATG* genes specifically involved in this pathway are not conserved; in contrast, those genes that are also needed for autophagy are highly conserved [61]. However, selective types of autophagy clearly take place in higher eukaryotes, including mitophagy and pexophagy. The molecular machinery involved in these processes in mammalian cells has not been completely elucidated, but it is likely that the general mechanism is conserved. For example, receptors such as BNIP3L and BNIP3 function as receptors in mammalian mitophagy, whereas Atg32 carries out this function in yeast; BNIP3L and BNIP3 are not homologs of Atg32, but they are functional counterparts, supporting the concept of mechanistic conservation. Furthermore, most of the machinery for the Cvt pathway is also used for pexophagy and mitophagy, which, as noted above, take place in higher eukaryotes. This means that with regard to the Atg proteins, the apparent absence of the Cvt pathway in mammals may be viewed as a deficiency in the specific receptor Atg19, rather than a major difference between yeast and other eukaryotes.

Returning to the initial question regarding the origin of the Cvt pathway, one possibility is that the oligomeric structure of prApe1 or Ams1 is critical for stability and/or function. The size of the oligomeric form of these hydrolases would prevent translocation through the ER translocon, necessitating a vesicle-mediated import process. Also, Ams1 does not appear to be synthesized as a zymogen. Thus, it would be problematic for this hydrolase to traverse the secretory pathway along with other newly synthesized glycosylated proteins. These vacuolar hydrolases are likely required in large amounts when the cell is starved or when aggregated proteins or damaged organelles accumulate, and the synthesis of most vacuolar hydrolases increases substantially during starvation. Under these conditions, the efficient transport of these hydrolases as oligomers by means of a vesicle-mediated mechanism such as autophagy would be extremely efficient. It would seem reasonable for the cell to modify the autophagy pathway very slightly with the addition of a small number of specificity components to take advantage of the existing autophagy machinery and allow it to be used for various types of selective sequestration processes.
